# Anterior segment optical coherence tomography (AS‐OCT) assisted analysis of characteristics of graft dehiscence after Descemet membrane endothelial keratoplasty (DMEK) for failed penetrating keratoplasty

**DOI:** 10.1111/aos.17445

**Published:** 2025-01-21

**Authors:** Pratima Vishwakarma, Mert Mestanoglu, Veronika Welge‐Lüßen, Simona Schlereth, Claus Cursiefen, Björn Bachmann

**Affiliations:** ^1^ Department of Ophthalmology, Faculty of Medicine University of Cologne Cologne Germany; ^2^ S. M. Vishwakarma Memorial Eye Hospital Nagpur India; ^3^ CECAD, Cluster of Excellence University of Cologne Cologne Germany; ^4^ Center for Molecular Medicine Cologne (CMMC) University of Cologne Cologne Germany

**Keywords:** anterior segment optical coherence tomography, descemet membrane endothelial keratoplasty, graft dehiscence, graft failure, penetrating keratoplasty

## Abstract

**Purpose:**

To analyse anterior segment optical coherence tomography (AS‐OCT) parameters of graft dehiscence after Descemet membrane endothelial keratoplasty (DMEK) for graft failure post penetrating keratoplasty (PK).

**Methods:**

Retrospective evaluation of AS‐OCT images of 142 dehiscences post‐DMEK in 75 eyes. Dehiscences' size, depth, location, correlation with graft‐host interface (GHI) override and step at GHI were assessed.

**Results:**

The majority of patients were male (58.7%) and mean age was 67 ± 13.6 years. Multiple dehiscences were observed in 49.3% eyes. Rebubbling was required once in 72% and multiple times in 24% eyes. Among 142 dehiscences, crossing over GHI was noted in 53.5%. The median distance of peripheral edge of dehiscences from GHI was 0.21 mm. Steps at GHI were noted in 41.5% where 22.5% dehiscences with step crossed the GHI. For dehiscences crossing versus not crossing GHI, no significant difference was noted in median depth (*p* = 0.268) and size (*p* = 0.206). For dehiscences crossing over GHI with presence versus absence of step, median depth (*p* = 0.23) and size (*p* = 0.196) showed no significant difference. No significant difference was noted in dehiscences' median depth (*p* = 0.16) and size (*p* = 0.926) among different step sizes. Incidence of dehiscence with or without steps (*p* = 0.8853) and graded as per size of steps, showed no significant difference.

**Conclusion:**

Size and depth of dehiscence were not influenced by their crossing over GHI or the presence and size of steps. Dehiscences typically occurred in close vicinity to GHI, suggesting that DMEK graft should be placed 0.25 mm centrally from the prior PK interface, independent of GHI irregularities.

## 1 INTRODUCTION

There has been a recent increase in trend towards anterior and posterior lamellar keratoplasties instead of full‐thickness penetrating keratoplasty (PK), but still PK is required for several conditions where the corneal clarity is lost involving the entire corneal thickness (Flockerzi et al., 2024; Matthaei et al., 2017; Qureshi & Dohlman, 2023; Reddy et al., 2013). With the current surgical and medical management a success rate of around 80% after 2 years has been noted for corneal transplants, but eventually there are chances of endothelial graft rejection, endothelial cell loss and graft failure (Bohringer et al., 2010; Borderie et al., 2009; Cursiefen et al., 2005; Schrittenlocher et al., 2018).This can lead to loss of graft transparency which can be reversed mostly during the rejection episode with topical and systemic corticosteroids but require surgical intervention like repeat PK, DSAEK (Descemet stripping automated endothelial keratoplasty [EK]) or DMEK (Descemet membrane EK) if there is graft failure (Aboshiha et al., 2018; Costa et al., 2009; Kitzmann et al., 2012; Sangwan et al., 2005; Tandon et al., 2009; Wykrota et al., 2024; Yamazoe et al., 2013). In cases where there is absence or minimal scarring of the stroma and absence of high or irregular corneal astigmatism, EK methods like DSAEK and DMEK are preferred over repeat PK in view of lesser chances of intraoperative complications like suprachoroidal haemorrhage, lens expulsion or intracameral bleeding and post operative complications like graft failure, loosening of sutures, persistent epithelial defects or high astigmatism and lower rates of immune reactions (Flockerzi et al., 2018; Pasari et al., 2019; Schrittenlocher et al., 2020; Wykrota et al., 2024). DMEK when compared to DSAEK have earlier and better visual recovery but has higher chances of post operative graft dehiscence which may require further procedures like rebubbling (Flockerzi et al., 2018; Schrittenlocher et al., 2020; Tourtas et al., 2012). Recently several studies have described good outcomes of DMEK with faster visual recovery and higher safety profile compared to repeat PK in cases with graft failure following PK (Einan‐Lifshitz et al., 2018; Güell et al., 2019; Heinzelmann et al., 2017; Hos et al., 2021; Pierne et al., 2019; Safadi et al., 2020; Schrittenlocher et al., 2020; Steindor et al., 2022; Wykrota et al., 2024). However, there is a high rate of rebubbling of up to 49%, which may increase the likelihood of graft failure or other complications (Einan‐Lifshitz et al., 2018; Safadi et al., 2020). This makes it imperative to find the causative factors leading to graft detachment. In our study, we have analysed the characteristics of the dehiscence with the help of anterior segment optical coherence tomography (AS‐OCT). With this study we aim to determine the cause of the graft dehiscence in relation to the characteristics of the DMEK graft, prior PK graft and interface between the host and the prior PK graft.

## 2 MATERIALS AND METHODS

This is a retrospective analysis of medical records of 75 patients who presented with graft dehiscence after DMEK in cases with post PK graft failure due to endothelial rejection. The data were obtained from the prospective Cologne DMEK Database (Schrittenlocher et al., 2017). The study period was from 1st January 2012 to 31st December 2019, adhering to the tenets of the Declaration of Helsinki. AS‐OCT (slit‐lamp‐OCT or Spectralis OCT, Heidelberg Engineering, Heidelberg, Germany) was performed in the immediate post‐operative period for all cases. Parameters of the dehiscence were assessed with the help of the Heidelberg Eye Explorer software and measurements were done using the integrated measuring tool (Heidelberg Engineering) in terms of the location, size and depth of dehiscence, distance of the dehiscence from the center of the cornea, override of dehiscence over the previous PK graft‐host interface (GHI), distance of edge of dehiscence from the GHI, presence of step (mismatch of edge of the host or prior PK graft at the posterior GHI) and size (vertical mismatch) of step. Correlation between the size and depth of the dehiscence was assessed in relation to the presence of DMEK scroll override over the prior GHI and to the presence of step at the GHI.

For statistical analysis, data was noted to be quantitative with non‐normal distribution as median with 25th and 75th percentiles (interquartile range). The data normality was checked by using Shapiro–Wilk test. The cases in which the data was not normal, non‐parametric tests were used. The association of the variables which were quantitative and not normally distributed in nature were analysed using Mann–Whitney Test (for two groups) and Kruskal Wallis test (for more than two groups). The presentation of the categorical variables was done in the form of number and percentage (%). For comparison of any variables which were qualitative in nature, Chi‐Square test was used. If any cell had an expected value of less than 5 then Fisher's exact test was used. The data entry was done in the Microsoft Excel spreadsheet and the final analysis was done with the use of Statistical Package for Social Sciences (SPSS) software, IBM manufacturer, Chicago, USA, version 25.0. For statistical significance, *p* value of less than 0.05 was considered statistically significant.

## 3 RESULTS

Overall, 142 dehiscences in 75 eyes were assessed. Among these, 38 eyes (50.7%) had dehiscence in one location while 37 eyes (49.3%) had multiple areas of dehiscences (2 areas in 20 eyes, 3 in 9 eyes, 4 in 4 eyes, 5 in 3 eyes and 6 in 1 eye). There were 44 (58.7%) males and 31 (41.3%) females in the study group. Average age at presentation was 67 ± 13.6 years. Pre‐existing visual limitations were absent in 18 patients. Prior surgeries required in these patients apart from PK were strabismus surgery (*n* = 2), dacrocystorhinostomy (*n* = 1), vitrectomy (*n* = 6), cyclophotocoagulation (*n* = 5), trabeculectomy (*n* = 3), panretinal photocoagulation for diabetic retinopathy (*n* = 1), fine needle diathermy (*n* = 1), corneal crosslinking (*n* = 1), and glaucoma implants (*n* = 4). Number of previous PK was 1 in 50 cases, 2 in 19 cases, 3 in 2 cases, 4 in 3 cases, and 5 in 1 case. Prior to DMEK, eyes were phakic in 14 cases, aphakic in 2 cases and pseudophakic in 59 cases. DMEK graft size was 7 mm in 4 cases (5.3%), 7.5 mm in 6 cases (8%), 8 mm in 64 cases (85.3%) and 10 mm in 1 case (1.3%). DMEK graft was attached with air tamponade in 24 eyes and 20% SF6 gas in 51 eyes. Rebubbling was required in 54/75 patients (72%) with 36/75 (48%) requiring rebubbling once, 13/75 (17.3%) twice, 4/75 (5.3%) thrice, and 1/75 (1.3%) requiring rebubbling 4 times for complete graft attachment. Median BCVA (best corrected visual acuity) as logMAR before DMEK was 1.3 (IQR, 0.7–1.6). Median BCVA at 1 week, 1 month, 3 months, 6 months, 1 year, 2 years, and 3 years were 1.65 (IQR, 1–2.3), 1 (IQR, 0.65–1.35), 0.8 (IQR, 0.47–1.3), 0.8 (IQR, 0.45–1.3), 0.7 (IQR, 0.4–1.2), 0.7 (IQR, 0.3–1), and 1.3 (IQR, 0.3–1.7), respectively.

**FIGURE 1:**
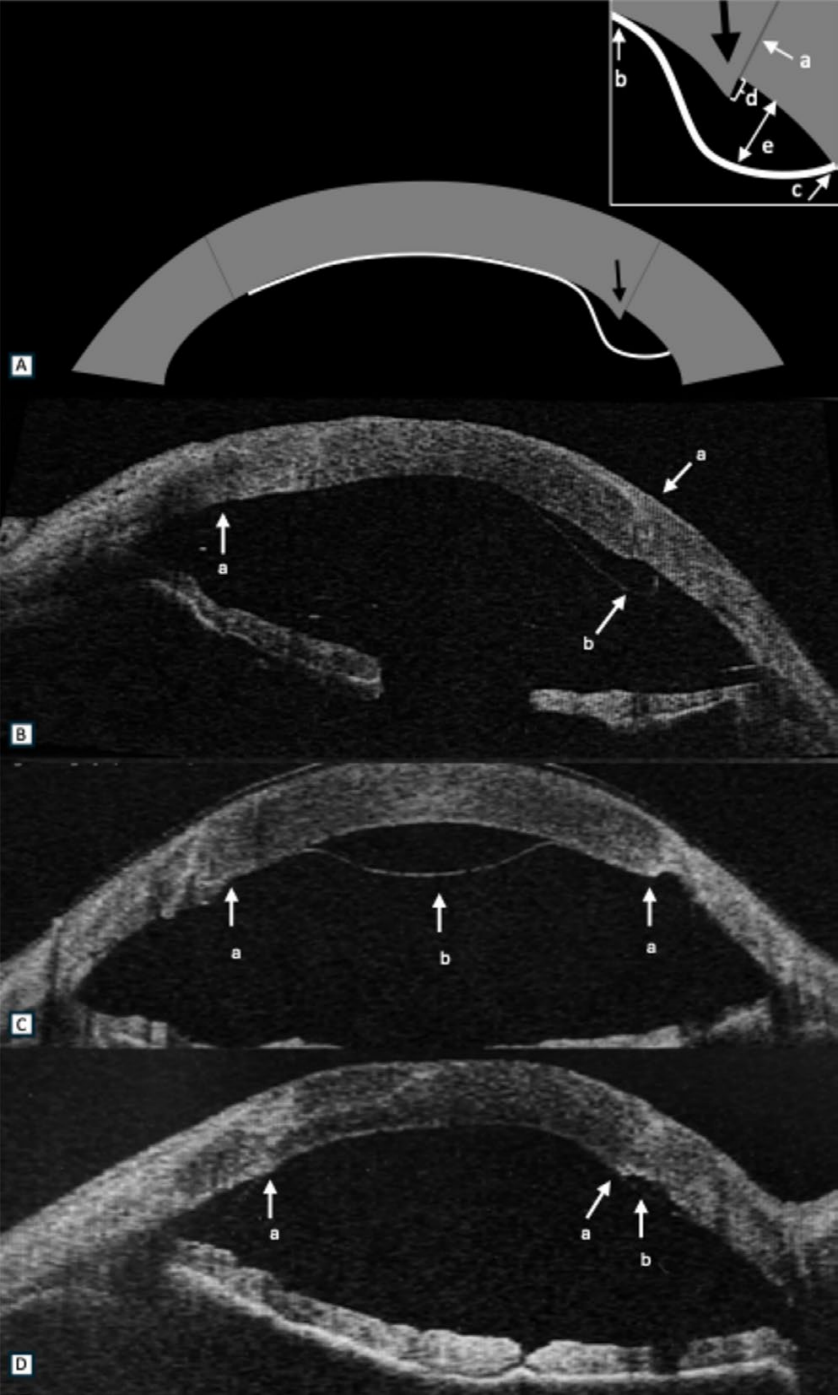
Anterior segment optical coherence tomography images of cases with graft dehiscence after DMEK (Descemet membrane endothelial keratoplasty) post PK (penetrating keratoplasty). (A) Illustrative diagram showing dehiscence crossing the graft host interface (GHI) with presence of step i.e. the posterior irregularity at the GHI (black arrow). Inset showing magnified image of the area of dehiscence showing prior PK GHI (a), central edge (b), and peripheral edge of dehiscence (c), size of step (d), and the depth of dehiscence (e). (B) Dehiscence (b) crossing the GHI (a). (C) Dehiscence not crossing the GHI (a) and located centrally (b). (D) Dehiscence not crossing the GHI (a) and located peripherally (b).

Immediate post‐operative AS‐OCT images were analysed for all 142 dehiscences of the 75 eyes. (Figure 1) Dehiscence was noted to be crossing over the GHI in 53.5% (76/142) occasions while it did not cross the GHI in 46.5% (66/142). Dehiscences were noted in superior quadrant in 5/142 (3.5%), temporally in 60/142 (42.3%), inferiorly in 13/142 (9.2%), nasally in 52/142 (36.6%) and complete dehiscence was noted in 12/142 (8.5%). Among the 142 dehiscences, steps at the GHI were noted in 59/142 (41.5%) of which 32/142 (22.5%) had dehiscence passing over the GHI. Median size of dehiscences was noted to be 2.1 mm (IQR, 1.4–3.2) and depth was noted to be 0.3 mm (IQR, 0.2–0.5).

Median distance of the peripheral edge of dehiscences to the GHI was 0.21 mm (IQR −0.24 to 0.6), from the central edge of the dehiscence to the GHI was −1.88 mm (IQR −3.33 to −1.25) and from the graft edge to the GHI was 0.39 mm (IQR 0–0.81) (negative values denoting distance from interface towards the centre of cornea and positive value from interface towards the periphery of cornea).

For the dehiscences crossing over the GHI, the median distance of the GHI from the edge of the dehiscence towards the center was 1.49 mm (IQR, 0.85–2.81), towards the periphery was 0.57 mm (IQR, 0.37–0.9) and the distance of the GHI from the peripheral edge of the graft was 0.6 mm (IQR, 0.37–0.96).

For the dehiscences not crossing over the GHI, 64/66 (96.97%) dehiscences were central that is, within the GHI and 2/66 (3.03%) dehiscences were peripheral that is, beyond the GHI. For these, the overall median distance of the GHI from the edge of the dehiscence towards the center was 2.28 mm (IQR, 1.82–3.47) and towards the periphery was 0.27 mm (IQR, 0–0.60). Though the dehiscences did not cross over the GHI, in 21/66 (31.82%) dehiscences the graft crossed over the interface and the median distance of the GHI from the peripheral edge of the graft crossing over the interface was 0.84 mm (IQR, 0.5–1.23) and for those not crossing over the GHI (*n* = 45/66, 68.18%) was 0.08 mm (IQR, 0–0.42).

The depth and size of the dehiscences were compared among those dehiscences crossing over the GHI and those not crossing over the GHI. The median depth of the dehiscences crossing over the GHI was 0.32 mm (IQR, 0.25–0.42) and for those not crossing over the GHI was 0.31 mm (IQR, 0.23–0.47) (*p* = 0.268, Mann–Whitney test). The median size of the dehiscences crossing over the GHI was 2.14 mm (IQR, 1.55–3.38) and for those not crossing over the GHI was 1.83 mm (IQR, 1.43–2.79) (*p* = 0.206, Mann–Whitney test).

**FIGURE 2:**
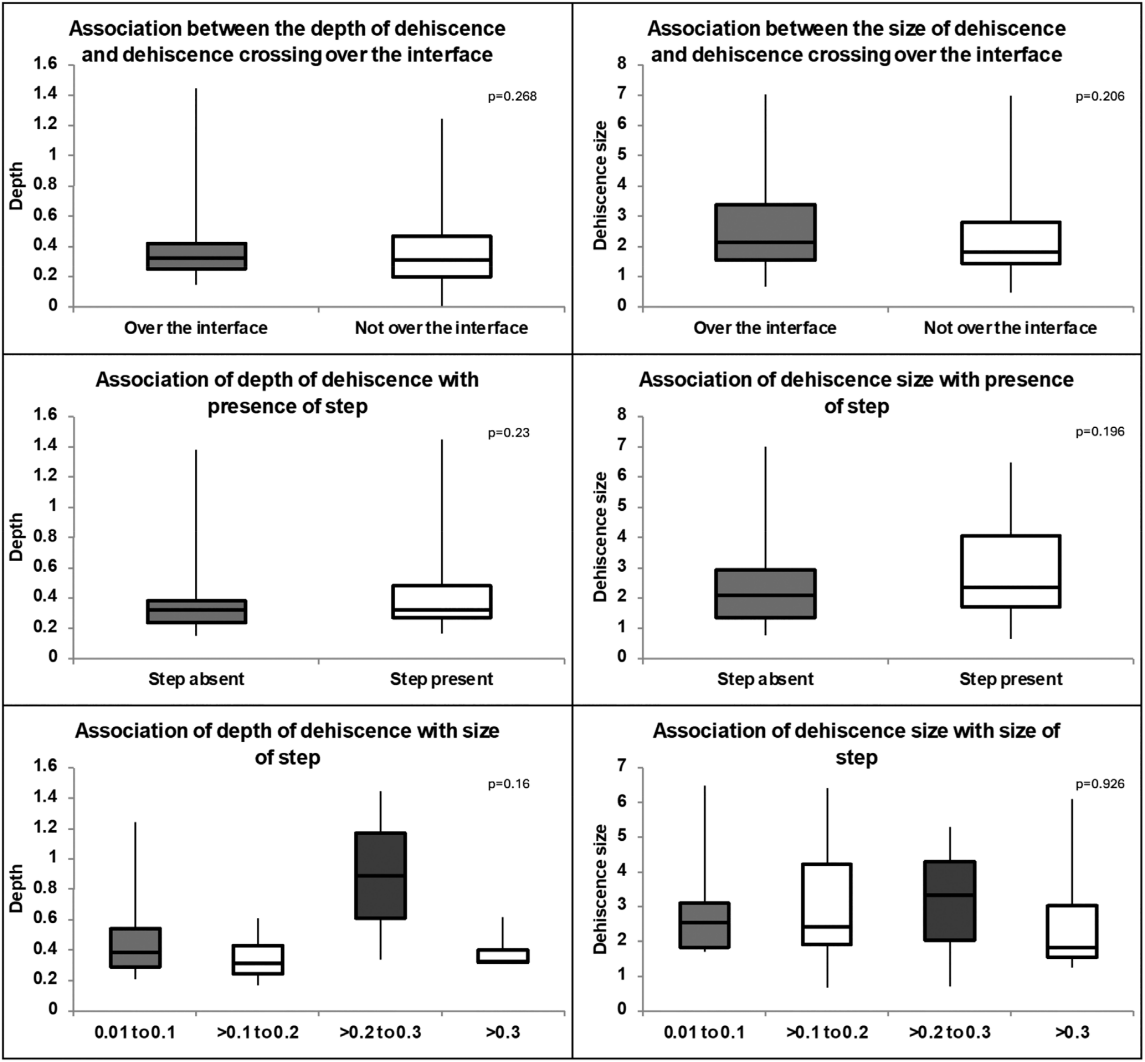
Association of depth and size of dehiscence to whether the dehiscence is crossing over the prior PK graft‐host interface and to the presence of step and its size. (non‐parametric variable, Box‐whisker plot).

The depth and size of the dehiscences with presence or absence of step at the GHI were compared for those dehiscences crossing over the GHI. The median depth of the dehiscences with presence of step was 0.32 mm (IQR, 0.24–0.38) and for those without step was 0.33 mm (IQR, 0.27–0.48) (*p* = 0.23, Mann–Whitney test). The median size of the dehiscences with presence of step was 2.09 mm (IQR, 1.37–2.93) and for those without step was 2.34 mm (IQR, 1.72–4.06) (*p* = 0.196, Mann–Whitney test). The depth and size were further compared based on the size of the step. For size of the step from 0.01 to 0.1 (*n* = 6), >0.1 to 0.2 (*n* = 19), >0.2 to 0.3 (*n* = 3) and >0.3 mm (*n* = 4), the median depth of the dehiscence was 0.38 mm (IQR, 0.288–0.54), 0.32 (IQR, 0.243–0.429), 0.89 (IQR, 0.612–1.168), 0.33 (IQR, 0.319–0.403), respectively (*p* = 0.16, Kruskal Wallis test) and size of dehiscence for was 2.54 (IQR, 1.844–3.097), 2.42 (IQR, 1.898–4.233), 3.34 (IQR, 2.027–4.309), 1.83 (IQR, 1.559–3.033), respectively (*p* = 0.926, Kruskal Wallis test). (Figure 2) The incidence of dehiscence with presence of steps as well as graded as per the size of steps, was compared with those without steps, details of which are given in Table 1.

**TABLE 1:** Comparison of Descemet membrane endothelial keratoplasty (DMEK) graft dehiscences with or without those crossing the graft‐host interface (GHI) of the prior penetrating keratoplasty with presence of step versus those without step.

		Dehiscences with step (n)	Dehiscences without step (n)	*p* value
Overall	Crossing over GHI	32	44	0.8853^a^
	Not crossing over GHI	27	39	
Step size: 0.01 to 0.1 mm	Crossing over GHI	6	44	0.5015^b^
	Not crossing over GHI	3	39	
Step size: >0.1 to 0.2 mm	Crossing over GHI	19	44	0.1132^a^
	Not crossing over GHI	8	39	
Step size: >0.2 to 0.3 mm	Crossing over GHI	3	44	0.1202^b^
	Not crossing over GHI	9	39	
Step size: >0.3 mm	Crossing over GHI	4	44	0.35^b^
	Not crossing over GHI	7	39	

Abbreviation: n, number of dehiscences.

^a^ Chi square test.

^b^ Fisher's test.

## 4 DISCUSSION

DMEK has proven to be a good surgical option for treatment of graft failure post PK, although recurrent graft detachment has been recognized (Einan‐Lifshitz et al., 2018; Safadi et al., 2020). In a study by Safadi et al., pre‐operative AS‐OCT was assessed to detect the irregular bulging scars at the GHI considering that it can interfere with graft attachment. They suggested to either excise the bulging tissue or else undersize the DMEK graft so that it does not extend beyond the PK GHI (Safadi et al., 2020).

In our study, to analyse the anatomical cause of DMEK graft dehiscence in post PK cases, we assessed the immediate post‐operative ASOCT images of the graft dehiscence in 75 eyes where DMEK was done after endothelial graft failure post PK. Among the 75 cases, a male preponderance was noted (58.7%) with average presentation in the 7th decade of life (67 ± 13.6 years). Most eyes were pseudophakic (78.7%). Multiple areas of dehiscence were noted in 37 eyes (49.3%) thus amounting to a total of 142 dehiscence among the 75 eyes. As opposed to prior study where an inferior graft detachment was more common after DMEK, a recent study by Siebelmann et al. showed no preference in the location of the detachments (Siebelmann et al., 2018; Siebelmann et al., 2021). In our study too, upon analysis of ASOCT images in DMEK post PK, there was no inferior predilection for graft detachment.

Rebubbling rates were higher in these cases (72%) with requirement for multiple rebubbling in 24% cases (18/75) when compared to a prior study where DMEK was done in our institute in cases without any prior PK (32.3%) (Siebelmann et al., 2021). In a study by Steindor et al., among the 11 eyes only 2 (18.2%) required rebubbling post DMEK after PK despite oversizing the DMEK grafts compared to the prior PK graft (Steindor et al., 2022). Another study by Pasari et al., showed increased rates of rebubbling in cases post DMEK after prior failed PK where the DMEK graft size was bigger than that of the prior PK graft (53%) compared to when it was of the same size (27%) or smaller (33%) (Pasari et al., 2019). In a study by Pierné et al., rebubbling rate was 50% despite graft undersizing by 0.25 mm compared to the prior PK graft. They considered suboptimal graft sizing, graft decentration, lesser intra‐operative air tamponade and incomplete descemetorhexis of the prior PK graft as the possible causative factors for the graft dehiscence (Pierne et al., 2019).

In this study we wanted to assess the relationship between various parameters of the dehiscence to the prior PK graft, GHI and presence of step at the interface. Upon comparing the median depth of the dehiscences crossing over the GHI with those not crossing over the GHI, no significant difference was noted (*p* = 0.268). Also, the median size of the dehiscences crossing over the GHI versus those not crossing over the GHI showed no significant difference (*p* = 0.206). Thus, the size and depth of the dehiscence were not affected by the crossing of the dehiscence over the GHI.

**FIGURE 3:**
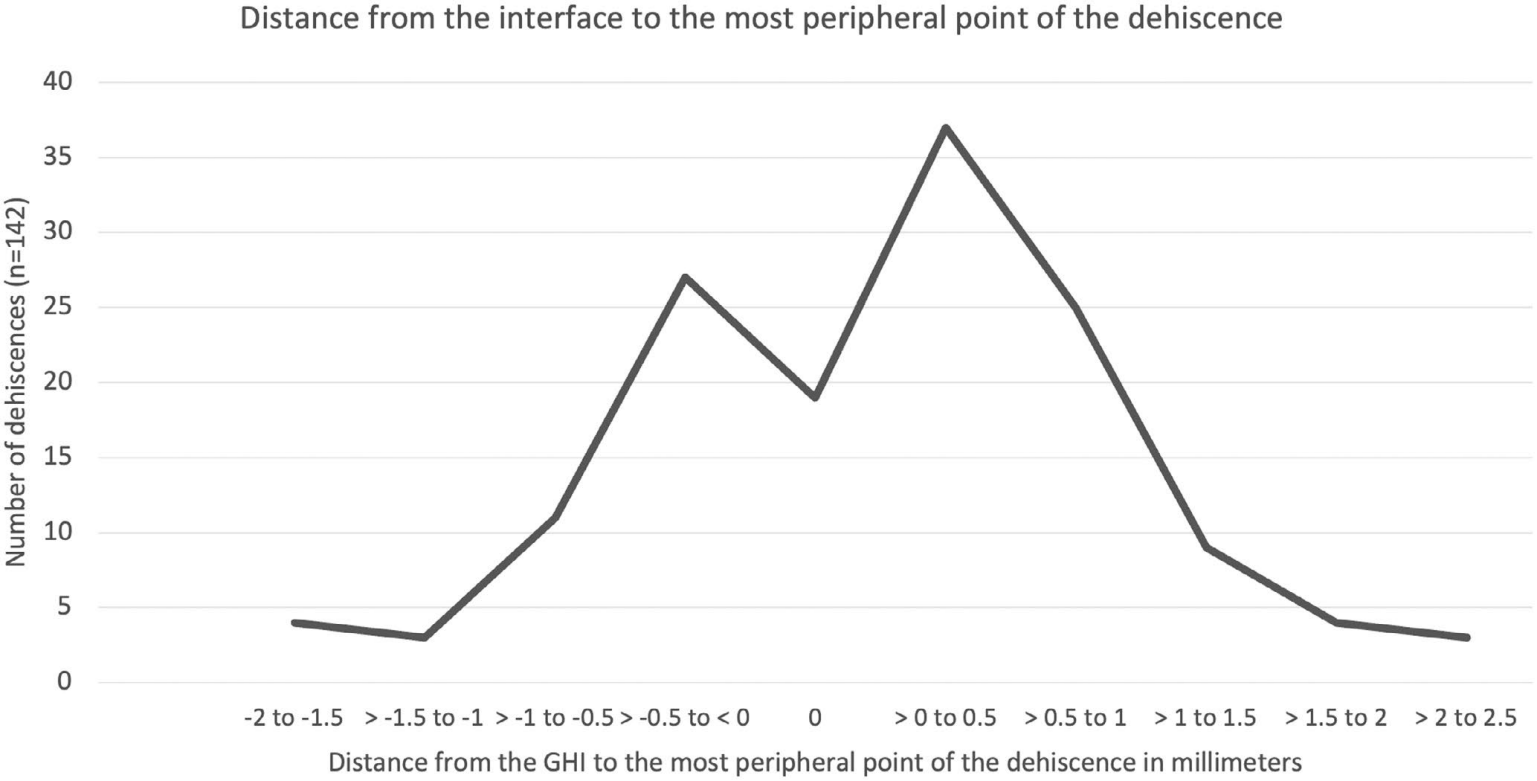
Distance from the interface to the most peripheral point of the dehiscence in millimetres (negative values denotes the distance from interface towards the center of cornea, 0 denotes the interface and positive value denotes the distance from the interface towards the periphery).

The median distance of the peripheral edge of the dehiscence from the GHI was noted to be 0.57 mm (IQR, 0.37–0.9) for the dehiscences crossing over the GHI and 0.27 mm (IQR, 0–0.60) for dehiscences not crossing over the GHI. Overall, median distance of the peripheral edge of dehiscences from the GHI was 0.21 mm (IQR −0.24 to −0.6). (Figure 3) Thus, a close proximity could be noted between the peripheral edge of the dehiscences and the prior PK GHI.

Steps along the PK GHI can lead to graft lift‐off after sequential DMEK leading to graft dehiscence (Wykrota et al., 2024). Upon comparison between the median depth of the dehiscences crossing over the GHI with presence and absence of step, no significant difference was noted (*p* = 0.23). The median size of the dehiscences crossing over the GHI with presence or absence of step showed no difference (*p* = 0.196). Also, no significant difference was noted upon comparing the median depth (*p* = 0.16) and size (*p* = 0.926) of dehiscences among different sizes of the step. The incidence of the dehiscences crossing over the GHI and those not crossing over the GHI in presence of step versus in absence of step was compared; no significant difference was noted overall and when graded as per the size of steps (Table 1). Thus, in our study, presence of a step and its size did not seem to affect the depth, size and incidence of the dehiscence. This indicates that though steps may not have impact on the dehiscence parameters, other minor irregularities, which occur in almost all interface areas after PK, have a considerable influence on the detachment of the DMEK graft. It can be assumed that the irregularity of the posterior corneal surface decreases with distance from the PK interface, which is reflected in the lower number of dehiscences with greater distance from the interface.

Limitations of this study is its retrospective nature. Moreover, horizontal cross‐sectional AS‐OCT images were assessed, which means that radial measurements of the dehiscences was not possible. This represents the greatest limitation of our investigation, as the horizontal measurements can overestimate the minimum distance of the dehiscence to the interface. This would, however, mean that the effect of dehiscences near the PK interface demonstrated by analysis of our horizontal cross‐sectional images would be even more pronounced with radial measurements. A further limitation of our analysis is that many patients had prior surgeries done elsewhere and so information regarding the size of prior PK graft, BCVA prior to PK graft failure and prior complications are not known in all cases. Additionally, pre‐operative AS‐OCT images and data on the total time of tamponade were unavailable for most patients.

In conclusion, this study shows that the dehiscences are a very common phenomenon after DMEK for failed previous PK. Also, dehiscences are commonly located across the GHI but unexpectedly the parameters of the prior PK (like the relation of the dehiscence to the prior GHI and the irregularities at the edge of the prior graft) do not seem to affect the size and depth of dehiscence.

Therefore, we need to further evaluate the other causes for dehiscences like issues with tissue adhesion in the GHI, altered fluid currents in the GHI region, graft centration, graft size, size of descemetorhexis, duration and pressure of gas tamponade.

Our evaluation suggests that the edge of the DMEK graft should have a distance to the PK interface, independent of the regularity of the PK GHI. The edge of the DMEK graft should be at least 0.25 mm away from the prior PK GHI (0.5 smaller than the PK graft) as we have noted that the median distance of the peripheral edge of dehiscences from the GHI to the periphery was 0.21 mm.

## FUNDING INFORMATION

Financial support was provided by the German Research Foundation (SFB 1607; www.crc1607.de; MM, SS, CC, BB).

## CONFLICT OF INTEREST STATEMENT

There is no conflict of interest.
